# From Iron Chelation to Overload as a Therapeutic Strategy to Induce Ferroptosis in Leukemic Cells

**DOI:** 10.3389/fonc.2020.586530

**Published:** 2020-09-18

**Authors:** Eric Grignano, Rudy Birsen, Nicolas Chapuis, Didier Bouscary

**Affiliations:** ^1^Université de Paris, Institut Cochin, CNRS UMR8104, INSERM U1016, Paris, France; ^2^Assistance Publique-Hôpitaux de Paris, Centre-Université de Paris, Service d'Hématologie clinique, Hôpital Cochin, Paris, France; ^3^Assistance Publique-Hôpitaux de Paris, Centre-Université de Paris, Service d'Hématologie biologique, Hôpital Cochin, Paris, France

**Keywords:** acute myeloid leukemia, iron, reactive oxygen species, ferroptosis, ferritinophagy

## Abstract

Despite its crucial importance in numerous physiological processes, iron also causes oxidative stress and damage which can promote the growth and proliferation of leukemic cells. Iron metabolism is strictly regulated and the related therapeutic approaches to date have been to restrict iron availability to tumor cells. However, since a new form of iron-catalyzed cell death has been described, termed ferroptosis, and subsequently better understood, iron excess is thought to represent an opportunity to selectively kill leukemic cells and spare normal hematopoietic cells, based on their differential iron needs. This review summarizes the physiology of iron metabolism and its deregulation in leukemia, the known ferrotoposis pathways, and therapeutic strategies to target the altered iron metabolism in leukemia for the purposes of initiating ferroptosis in these cancer cells.

## Introduction

Iron is a crucial nutrient for the enzymes that are involved in ATP production (mitochondrial chain complex), DNA synthesis (ribonucleotide reductase), oxygen transport, antioxidant defense (peroxidase and catalase), oxygen sensing factors (hypoxia-inducible factor – HIF – and prolyl hydroxylases), and many others. The ability of iron to gain and lose electrons between its oxidized Fe^3+^-ferric*-* and Fe^2+^-ferrous*-* forms enables it to participate in free radical-generating reactions. Among these processes is the Fenton reaction, in which a ferrous iron donates an electron to hydrogen peroxide to yield hydroxyl radicals, thereby inducing highly reactive oxygen species (ROS) (1). The aberrant accumulation of iron and subsequent excess ROS levels generate oxidative stress, induces damage to DNA, proteins or lipids and even causing cell death. Significantly, these oxidative effects of iron can contribute to oncogenesis and iron is thought to be essential for the development of cancer (2).

Acute myeloid leukemia (AML) results from oligoclonal proliferations arising from the transformation of immature myeloid hematopoietic cells, characterized by a differentiation blockage and acquisition of a survival advantage and proliferation gain. AML displays high heterogeneity at the phenotypic, genetic and molecular levels (3). Recurrent molecular abnormalities resulting from mutations or translocations define the prognostic subgroups of AML, which vary in their sensitivity to conventional treatments such as chemotherapy and bone marrow transplantation (4). The prognosis for AML remains largely poor but new therapeutic strategies are emerging, stemming from better pathophysiological knowledge. Lastly, the association of the BCL2-BH3 mimetic venetoclax and demethylating agents (azacytidine or decitabine) directly targets the leukemic stem cell (LSC) compartment via oxidative metabolism perturbation and has shown compelling effectiveness in elderly patients with AML (5, 6).

To enable growth, leukemic cells exhibit an increased iron demand compared with normal cells (7). Accumulating evidence indicates that targeting iron homeostasis can induce differentiation and apoptosis in some leukemic cells (8–10), and these studies almost exclusively focused on intra-leukemic iron depletion by chelating agents. However, this iron dependency may also render cancer cells more vulnerable to iron-mediated cell death, referred to as ferroptosis (11), in which iron metabolism plays a key role. Hence, strategies aimed at targeting leukemic cells by increasing the intracellular iron pool could constitute a new therapeutic approach in AML. We therefore highlight the phenomenon of iron metabolism deregulation in AML in this review and focus on the opportunity of targeting this process to improve AML treatment.

## Iron Metabolism: An Overview

Iron homeostasis is a highly tuned process. Dietary iron (predominantly in the form of Fe^3+^) is absorbed in the duodenum via divalent metal transporter 1 (DMT1) after the action of a ferric reductase, duodenal cytochrome b (Dcytb), which converts Fe^3+^ to Fe^2+^. Alternatively, heme iron is imported into enterocytes through an unknown mechanism and is thereafter, degraded by heme oxygenase 1 (HO-1), liberating Fe^2+^. Iron exits the basolateral surfaces of the enterocytes across the sole iron efflux pump yet known, ferroportin (FPN), along with hephaestin which oxidizes Fe^3+^ to Fe^2+^, and then is loaded onto transferrin (Tf). The holo-transferrin complex Tf – Fe^3+^ circulates in the plasma to deliver iron to its sites of utilization, mostly to the bone marrow to enable erythropoiesis. After binding to transferrin receptor 1 (TFR1), the Tf-Fe^3+^/TFR1 complex is subsequently taken into cells by receptor-mediated endocytosis, reduced to Fe^2+^ by the six-transmembrane epithelial antigen of prostate 3 (STEAP3), and transported into the cytosol by DMT1. The Tf/TFR1 complex is finally recycled to the cell surface. Recycling from senescent red blood cells by macrophages is by far the main source of iron, whereas dietary intake is marginal and compensates for iron losses only. The released Fe^2+^ constitutes the labile iron pool (LIP) and is either stored in ferritin (FTH) or used for metabolic needs, or even exported into the circulation via FPN after being re-oxidized by ceruloplasmin (12).

The predominant pathway for iron output into the cytosol from ferritin is controlled by nuclear receptor coactivator 4 (NCOA4)-mediated selective autophagy, whereby NCOA4 binds ferritin to traffic it to the lysosome where it is degraded and iron is then released for use by the cell (13). Non-stored cytoplasmic iron is either transported to mitochondria for heme synthesis or incorporated in Fe-S clusters. It can serve as a cofactor for several enzymes, such as prolyl hydroxylase 2 (PHD2), lipoxygenases (LOX), and others. At the systemic level, the iron balance is maintained via a strictly regulated absorption of dietary iron in the duodenum, which is mainly achieved by the ferroportin – hepcidin regulatory axis (14). The peptide hormone hepcidin, secreted primarily by hepatocytes, is the cardinal regulator of iron homeostasis. In conditions of excess iron, hepcidin binds to FPN and promotes its phosphorylation and subsequent lysosomal degradation in enterocytes and macrophages, thus blocking the delivery of iron into the circulation (15). At the cellular level, iron homeostasis is regulated at the post-transcriptional level by the binding of regulatory proteins IRP1 and IRP2 to the iron-responsive elements (IREs) within mRNAs. The IREs are hairpin structures located in 5′or 3′ untranslated regions (UTRs) of mRNAs and control either transcript stability or the initiation of translation. The role of the IRPs is to adapt, according to the cellular iron availability, and control the expression of proteins that regulate the import (TFR1, DMT1), storage (FTH), and export (FPN) of intracellular iron (16).

## Iron and Acute Myeloid Leukemia

The normal mechanisms of iron handling are altered in cancer cells in order to facilitate tumor growth. Evidence for iron overload involvement in promoting cancer came some time ago from *in vivo* experiments (17), epidemiological studies (18), and cancer susceptibility in hemochromatosis-affected patients (19), but the underlying biological mechanisms have been more precisely understood for a decade (2). In particular, because of the high red blood cell transfusion needs in cancer patients due to normal erythropoiesis impairment and chemotherapy-induced anemia, iron excess is a common finding in leukemic patients. The organism response consists of limiting iron bioavailability, referred to as “the withholding response,” and therapeutic developments to date that are based on iron metabolism have mostly focussed on enabling this withholding response.

Iron is crucial for normal hematopoiesis since TFR1 inhibition and subsequent iron depletion impair the proliferation and differentiation of hematopoietic precursor cells, and reduce the regeneration potential of hematopoietic stem cells (HSCs) (20). Notably however, excess iron and ROS catalytic production also promote the malignant transformation of HSCs through nicotinamide adenine dinucleotide phosphate oxidases (NOX) and the subsequent depletion of glutathione (GSH) (21). DNA damage and double-strand breaks induced by ROS in myelodysplastic syndromes, a pre-leukemic disorder, may foster the transformation process in AML (22). On the other hand, HSC aging is associated with increasing ROS production, impaired HSC self-renewal and regeneration (23), and decreased erythropoiesis trough growth differentiation factor-11 (GDF11) secretion (24). However, in particular stress contexts, ROS promote short-term HSC repopulation, motility and differentiation (25), thus acting as a kind of bone marrow microenvironment messenger. Moreover, in monocytic AML, blasts produce high amounts of ROS via NADP oxidase (NOX) and thereby induce apoptosis in adjacent NK cells, and in CD4 and CD8 T-cells, through poly-ADP-ribose polymerase-1 (PARP-1), and a subsequent ineffective anti-leukemic adaptive response (26). Iron overload also disrupts the T-lymphocyte Th1/Th2 ratio and promotes regulatory T-cell production (Treg) (27).

## Iron Dysregulation at the Cellular and Systemic Level

Iron markers of intake, storage and export are commonly concurring to iron overload. TFR1 expression, known as CD71, is generally increased in leukemic cells compared to normal counterparts and its level may be directly correlated to the degree of differentiation as AML, whether minimally differentiated or not, has the highest level of CD71 expression (28). The prognostic significance of this observation is debatable however as even if high levels of TFR1 are associated with complex karyotypes and other features such as c-kit receptor expression (CD117), the survival outcomes seem to be unaffected (29). Notably, transferrin receptor 2 (TFR2) is also upregulated in different AML subtypes such as AML1, AML2, and erythroleukemia (AML6) (30, 31), and TFR2 α-subtype expression may be positively associated with a favorable prognosis (32). TFR2 is mostly present at the surface of hepatocytes and erythroid cells and acts as an iron sensor and hepcidin modulator (33). Since both TFR1 and TFR2 increase intracellular iron, the former, by increasing iron uptake and the latter via hepcidin upregulation through HFE binding, the prognosis discrepancies between each isoform likely imply an iron-independent pathway for TFR2. It is worth mentioning also that other forms of non-transferrin bound iron may penetrate cancer cells, such as for instance via lipocalin 2 (LCN2), also referred as neutrophil gelatinase-associated lipocalin (NGAL), whose expression is detected in leukemic cells, but at a lower rates than in normal HSC counterparts (34, 35). LCN2 overexpression at the transcriptional level is associated with a better prognosis, especially in normal karyotype AML with FLT3 wild-type expression (36).

Regarding the iron storage counterpart, serum ferritin is also frequently increased in leukemic patients and is a pejorative factor in overall and relapse-free survival in chemotherapy-treated patients (37–40) as well as in patients undergoing allogeneic stem cell transplantation (41). Ferritin comprises 24 polypeptide subunits of heavy chain (FTH and light chain (FTL). In an inflammation context, FTH appears to be a NF-κB downstream effector that suppresses TNFα-driven apoptosis via the inhibition of Jun N-terminal kinase (JNK) (42). In AML patients, a gene expression signature associated with FTH overexpression encompasses NF-κB and pro-oxidant pathways, leading to chemotherapy resistance (37).

Dysregulation of the ferroportin-hepcidin axis is a common feature in AML, entailing reduced iron efflux. Notably, low ferroportin expression in AML seems to correlate with improved outcomes and greater chemotherapy sensitivity, and is consistently found in core binding factor (CBF) AML subsets (43). Hepcidin expression varies heterogeneously, depending on the amount of iron, and the degree of anemia and inflammation. In AML patients, positive correlations have been shown between hepcidin and both ferritin and IL-6, whereas a high EPO level and anemia are associated with lower levels of hepcidine (44). Notably however, despite the expected high levels of hepcidin under conditions of inflammation and overexpression of ferritin, balancing strategies to counteract anemia such as erythroblast secretion of erythroferrone (ERFE) can foster hepcidin downregulation (45). An example of this is seen in myelodysplastic syndromes with an SF3B1 mutation (46). In response to systemic limitations in iron availability, autocrine secretion of hepcidin to degrade FPN has been described in several cancer models, although not yet shown in AML (47–49).

## Therapeutic Strategies: Moving From Iron Chelation to Overload

The ability of tumor cells to fuel iron availability that enables sustained proliferation, ROS accumulation and evasion of adaptative host immunity has prompted research into iron chelation strategies. Moreover, such strategies became of paramount interest in the context of iterative blood transfusions in leukemic patients, in whom chronic iron overload leads to secondary hemochromatosis responsible for cardiac, hepatic, or endocrinal damage.

Hence, iron chelators such as deferoxamine (DFO) and deferasirox (DFX), by lowering LIP and hampering ROS production and iron-dependent enzymes such as ribonucleotide reductase (50–53), exert anti-leukemic activity. Interestingly, as mentioned earlier, FTH effects on the NF-κB pathway and the subsequent inhibition of both TNF-JNK signaling pathway-mediated apoptosis and iron chelators have been shown to restore JNK and mitogen-activated protein kinase (MAPK) pathways (54, 55). This ability, along with the use of other differentiating agents, may overcome the impaired cellular differentiation in AML (8). Likewise, iron chelators may interfere with hypoxia-induced pathways such as that controlled by HIF1α (56) or with the REDD1 – mammalian target of rapamycin (mTOR) pathway (57). Iron chelators can also sensitize leukemic cells to conventional chemotherapy drugs (58, 59) or to demethylating agents such as decitabine *in vitro* (60), although the data are conflicting (61).

Similarly, strategies aiming at modulating factors involved in iron metabolism such as TFR1, have shown promising efficacy since the early 1990's (62, 63). To date, several strategies have been developed for targeting TFR1, including the use of its natural ligand Tf, targeting peptides, anti-TFR1 monoclonal antibodies, and antibody fragments (scFv) (64). As an example of this, the A24 monoclonal antibody that competitively binds to TFR1, inhibits Tf binding to TFR1, thus leading to TFR1 endocytosis and its downregulation at the cellular surface, and has shown proven efficacy against T-NHL (65, 66). Such a strategy raises concerns however due to high dependency of erythropoiesis on TFR1 (67). As well, clinical studies have reported only moderate and transient efficacy against hematological malignancies (68), which can be partly explained by the induced immunogenicity (69). Hence, TFR1-targeted drugs have been developed as a delivery system, harnessing the overexpression of TFR1 in leukemic cells. For instance, TFR1-targeted lipid nanoparticles containing an antisense oligonucleotide (ASO) against the antiapoptotic protein Bcl-2 have been developed (70, 71), as well as TFR1 conjugated to the autophagy activator compound artemisinin (72). In addition, TFR1 has been conjugated to nanoparticles bearing chemotherapeutics that are designed to pass through the blood-brain barrier (73). Ferritin is also a potential candidate to shelter targeted drugs (74), such as doxorubicin (75), in order to produce a longer circulation half-life and a higher tumor uptake. Another intriguing drug delivery mechanism relies on a pro-drug strategy via trioxolane conjugation, which takes advantage of the higher amounts of iron in malignancies. In this mechanism, a trioxolane-conjugated drug will react with Fe^2+^ in the tumor microenvironment to activate drug release (76).

Despite the many clues that support iron chelation as an anti-tumor strategy in leukemia and the advances in this field, another way to exploit the increased iron needs in leukemic cells is to consider the vulberability that this creates. Indeed, cancer cells are far more susceptible to iron-catalyzed necrosis, referred to as ferroptosis. Hence, strategies aimed at increasing LIP in cancer cells may overcome the limited antioxidant defenses, thus entailing ferroptosis.

## Ferroptosis: a Recently Recognized Iron-Driven Form of Cell Death

**Figure 1:**
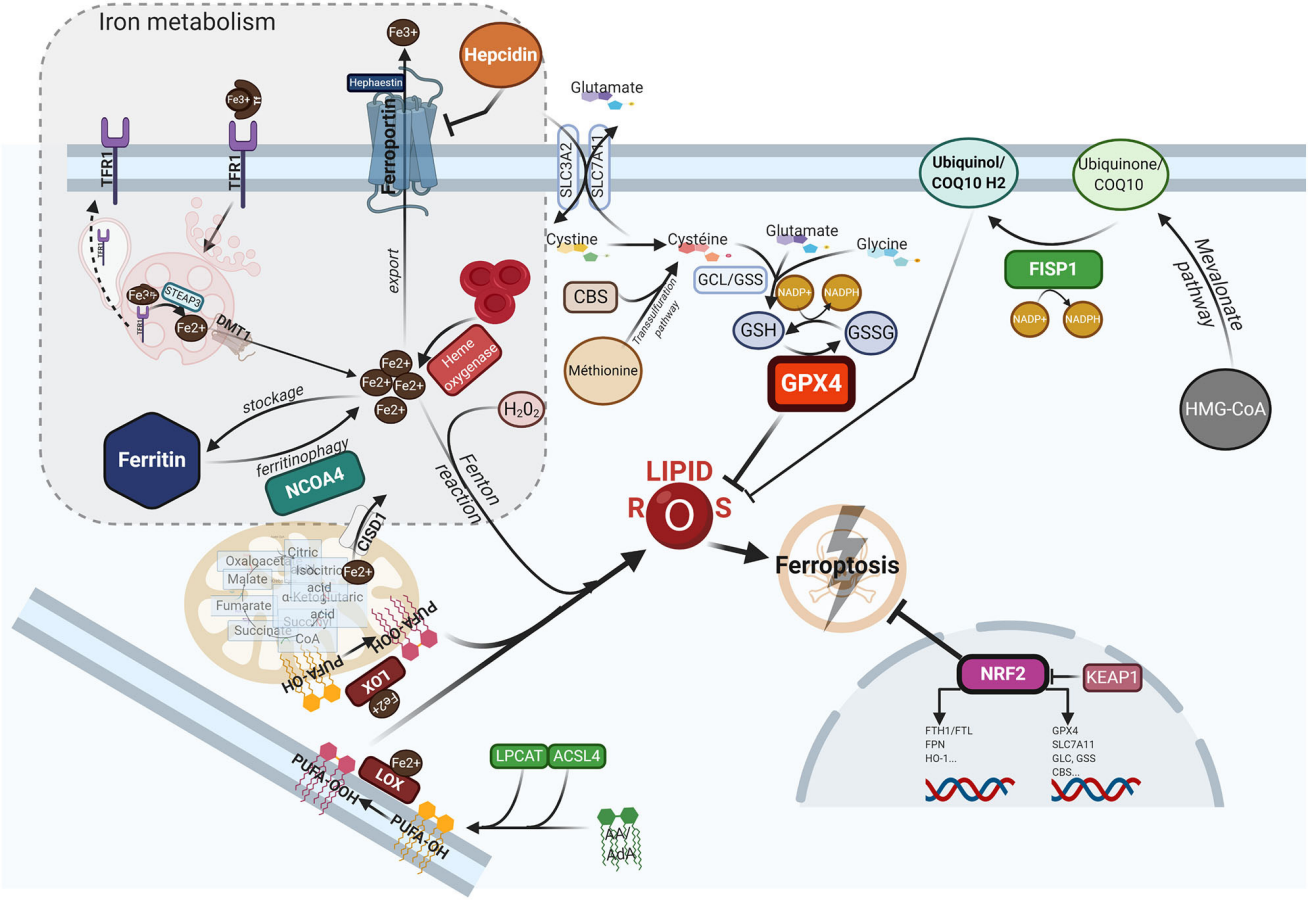
The ferroptotic cascade. Accumulation of free iron is a key initiator of ferroptosis. After loading onto Tf, the Fe^3+^/Tf complex binds to TFR1, and iron is thereafter released into endocytosis vesicles under lower pH conditions, where Fe^3+^ is reduced into the ferrous form Fe^2+^ under the action of ferrireductase STEAP3. Iron then exits into the cytosol via DMT1 to form the labile iron pool (LIP). The LIP represents a very slight per se, whereas most iron is either stored in FTH, either used as a cofactor for several iron-containing enzymes, or for the synthesis of heme. Excess iron is carried in the extracellular space through FPN after being reoxydized by hephaestin. FTH autophagic degradation, a process termed ferritinophagy, is mediated by NCOA4, a cargo receptor that binds to the heavy chain of FTH delivering it to the early-stage autophagosome, inducing the subsequent release of iron into the cytosol. Iron release from heme through HO-1 is another way to increase the LIP. Inside mitochondria, CISD1 modulates mitochondrial iron uptake and respiratory capacity and mitigates ferroptosis. The hormone hepcidin, mostly secreted by hepatocytes in the systemic circulation, triggers ferroportin lysosomal degradation, thus hindering iron exit. AA or AdA are key membrane phospholipids esterified by ACSL4 and LPCAT to generate PE-AA/ AdA, and further oxidized to phospholipid hydroperoxides (PE-AA/AdA-OOH) by LOXs. Free iron can also interact with ROS, specifically hydrogen peroxide, to form hydroxyl/peroxyl (LOH/LOOH) toxic radicals via the Fenton reaction. Therefore, iron can then abstract a hydrogen atom from PUFAs, forming a lipid radical which promptly reacts with oxygen to generate a lipid peroxide (PUFA-OOH). In a steady state, lipid peroxides and their degradation products are neutralized by GSH-based redox reactions. The xCT antiporter (consisting of two subunits SLC7A11 and SLC3A2) exports glutamine and imports cystine into the cell. Inside the cell, cystine is reduced to cysteine by cystine reductase and then GCL and GSS add L-glutamate and glycine respectively, to produce GSH. Another way to generate cysteine is via the trans-sulfuration pathway converting methionine in homocystein, and later cysteine by CBS. Many redox enzymes use GSH, including GPX4, which reduces reactive lipid peroxydes to their alcohol counterparts. Additionally, CoQ10, a byproduct of the mevalonate pathway, acts as a complemental RTA to mitigate ferroptosis. CoQ10 is reduced to CoQ1O-H2 by FSP1, enabling its activity. GPX4 and SLC7A11 are target genes of the master antioxidant regulator NRF2, as well as the enzymes contributing to GSH synthesis, GCL, GSS, and CBL. NRF2 additionally mediates iron metabolism by upregulating the transcriptional level of FTH, FPN, and HO-1. In the absence of redox stress, NRF2 is downregulated by the E3-ubiquitine ligase KEAP1. If the key redox regulator is genetically disrupted or pharmacologically inhibited, lipid peroxides and their degradation products accumulate, and thereby initiate ferroptosis through a yet unknown mechanism involving membrane destabilization, cytoskeletal changes, and cell death. AA, arachidonic acid; AdA, adrenic acid; ACSL4, acyi-CoA synthetase long chain family member 4; CBS, cystathionine-13-synthase; CISD1, CDGSH iron sulfur domain 1; CoQ10, coenzyme Q10/Ubiquinone-10; DMTl, divalent metal transporter 1; FSP1, ferroptosis-suppressor-protein 1; FPN, ferroportin; FTH, ferritin; GCL, glutamate-cysteine ligase; GPX4, glutathione peroxidase 4; GSH, glutathione; GSS, glutathione synthetase; HO-1, heme oxygenase 1; KEAP1, Kelch-like ECH-associated protein 1; LOXs, lipoxygenases; LPCAT, lysophosphatidylcholine acyltransferase; NCOA4, nuclear receptor coactivator 4; NRF2, nuclear factor (erythroid-derived 2)-like 2; PE, phosphatidylethanolamine; PUFA, polyunsaturated fatty acids; RTA, radical trapping agent; STEAP3, six-transmembrane epithelial antigen of the prostate 3; TFR1, transferrin receptor 1.

Ferroptosis is a form of programed cell death, that differs from apoptosis, necroptosis or pyroptosis in terms of the characteristic morphological changes i.e., mitochondria with decreased crista, condensed membranes and rupture of the outer membrane, and an intact nucleus (11). Biologically, ferroptosis is characterized by the iron-catalyzed peroxidation of polyunsaturated fatty acids (PUFAs) containing phospholipids (PLs), thereby producing lipid ROS. Arachidonic acid (AA) or adrenic acid (AdA) are key membrane phospholipids that are processed by acyl-CoA synthase 4 (ACSL4) and lysophosphatidylcholine acyltransferase 3 (LPCAT3) to produce their esterification and generate phosphatidylethanolamine (PE)-AA/AdA that undergoes further oxididation to phospholipid hydroperoxides (PE-AA/AdA-OOH) by the lipoxygenases (LOXs) (77). Excessive Fe^2+^ fuels electron-driven lipid peroxidation (LOOH) from an alkoxyl or an hydroxyl radical via the Fenton reaction in the presence of peroxide (H_2_O_2_). These reactions are not mutually exclusive and can operate together, especially since the LOXs are iron-containing enzymes (78). Meanwhile, lipid ROS can also be formed spontaneously by autoxidation enzyme-catalyzed processes (79). Once formed, lipid peroxides can diffuse across lipid bilayers in a self-feedback loop manner and compromise membrane integrity (80). To prevent the lethal accumulation of lipid ROS, several protective mechanisms are involved. One is glutathione peroxidase 4 (GPX4), which was shown to constitutively hydrolyze lipid hydroperoxides (LOOH) into inactive redox alcohol radicals (LOH), thereby protecting the cell from ferroptosis. GPX4 is a selenocysteine enzyme that uses glutathione (GSH) as a cofactor and can be covalently inhibited by molecules such as (1S,3R)-RSL3 (RSL3), or indirectly blocked by the small molecules FIN56 or FINO2 through a still elusive mechanism (81). Moreover, GSH synthesis requires the rate-limiting amino acid cysteine, which is imported in its oxidized form cystine from the extracellular space via the sodium-independent cystine/glutamate antiporter system ${\text{x}}_{\text{c}}^{-}$(consisting of two subunits SLC7A11 and SLC3A2). Inhibitors of system ${\text{x}}_{\text{c}}^{-}$, such as the oncogenic RAS-selective lethal small molecule erastin, sulfasalazine or sorafenib, subsequently triggers ferroptosis in different cellular contexts (11, 82). In contrast, several lipophilic radical-trapping agents (RTA), such as ferrostatin-1 (Fer-1), liproxtatin-1, or α-tocopherol, acting as ROS-lipid scavengers, are able to halt ferroptosis. Mitochondria can play a key role in ferroptosis depending on the context i.e., whilst they are crucial in the case of cysteine deprivation, they may not be required under conditions of GPX4 inhibition (83). Moreover, CDGSH iron sulfur domain 1 (CISD1), an iron-containing outer mitochondrial membrane protein, modulates mitochondrial iron uptake and respiratory capacity and mitigates ferroptosis (84). Interestingly, ferroptosis can also modulate tumorigenic immune defenses as immunotherapy-activation of CD8+ T cells by checkpoint inhibitors such as anti-PD1 or anti-CTLA4 enhances ferroptosis-specific lipid peroxidation in tumor cells via system ${\text{x}}_{\text{c}}^{-}$downregulation (85). Figure 1 summarizes the currently understood biology of ferroptosis, including its mechanisms and links with iron metabolism.

## Ferroptosis Relies on Iron Metabolism

A hallmark of ferroptosis is the requirement for iron, supported by the fact that iron chelation by deferoxamine (DFO) prevents the experimental induction of ferroptosis (Dixon, cell 2012), although it cannot do so once ferroptosis is launched (86). In addition, transferrin has been shown to be critical for this pathway since its deprivation dramatically decreases ferroptosis (gao, mol cell 2015), as well as the silencing of iron metabolism master regulator 2 (IREB2) (11). TFR1 endocytosis recycling inhibition by heat-shock protein beta-1 (HSPB1) overexpression and subsequent iron intake inhibition also alleviates ferroptosis (87). On the other hand, precluding TFR1 and FTH degradation by proteasome inhibitors such as bortezomib along with iron overload can cooperate to trigger ferroptosis (88). Likewise, DMT1 has been shown to be up-regulated upon ferroptosis induction (89). Ferritinophagy is another key component of the iron supply mechanism regulating ferroptosis. Indeed, autophagy inhibitors or NCOA4 cargo receptor genetic deletion suppress ferroptosis (90), whilst ferritin overexpression consistently mitigates ferroptosis in a neuronal model (91). Dihydroartemisinin (DHA), a semi-synthetic derivative of artemisinin known for its anti-tumor properties, was shown to induce ferritinophagy by regulating the activity of the AMP-activated protein kinase AMPK/mTOR/p70S6K pathway, thereupon triggering ferroptotic cell death (92). Interestingly, mitochondrial function is critical for iron metabolism and subsequent ferroptosis. Indeed, mitochondrial iron intake inhibition by CDGSH iron sulfur domain 1 (CISD1) overexpression markedly inhibits ferroptosis (84), as does the overexpression of iron-sulfur cluster assembly enzyme (ISCU), a mitochondrial protein which plays a crucial role in Fe-S cluster biogenesis (92).

## Targeting Iron in Leukemia to Trigger Ferroptosis

**Figure 2:**
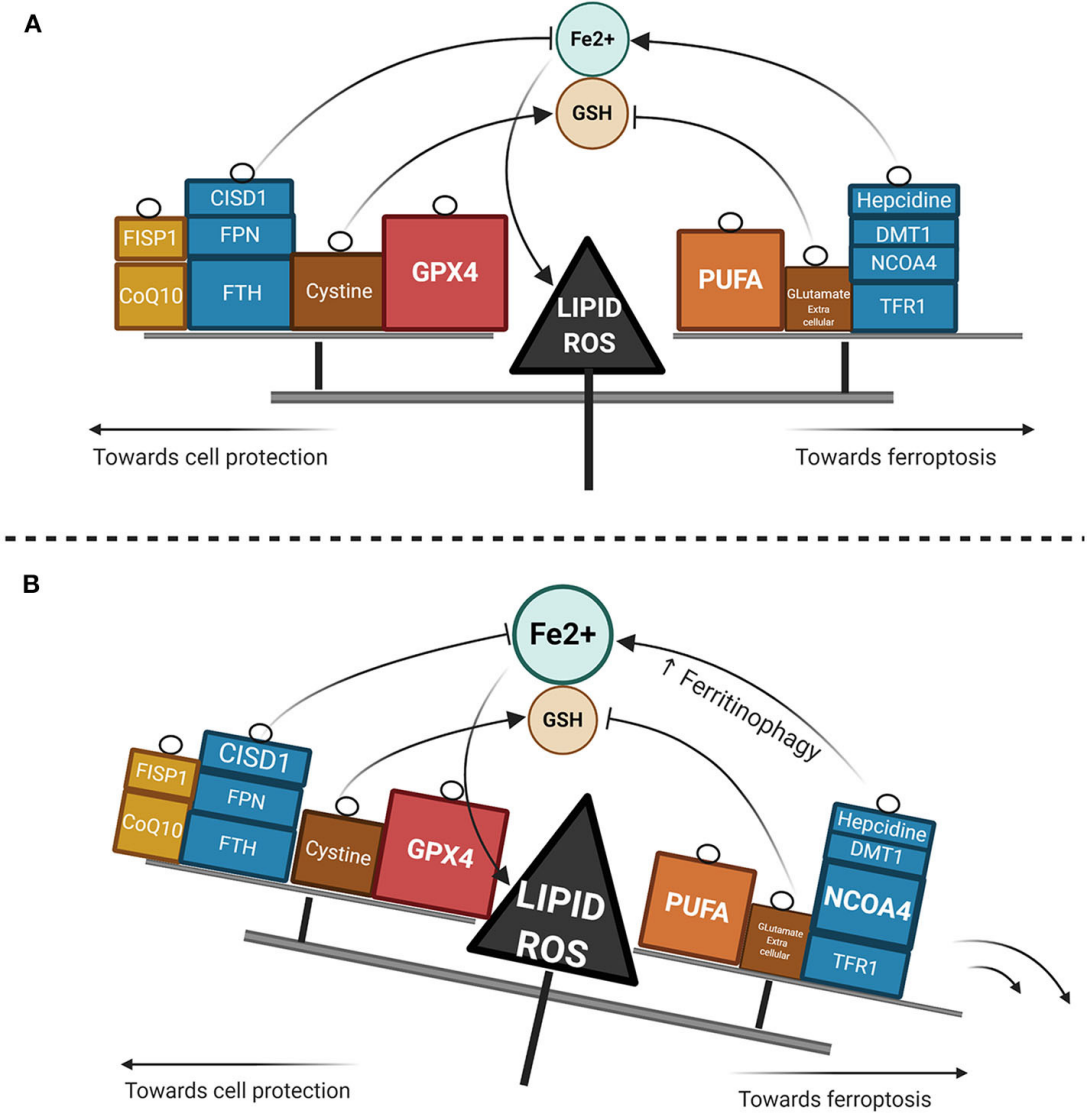
Lipid ROS homeostasis. **(A)** Schema showing enzymatic or substrate modulators of lipid ROS. The size of the box is proportional to the functional importance of the modulator. **(B)** Theorical imbalance of lipid ROS homeostasis, in this example caused by enhanced ferritinophagy through NCOA4 upregulation.

Ferroptosis has gained considerable interest as a potential widespread anti-cancer strategy, although most of the research thus far has been restricted to *in vitro* or murine *in vivo* models (93–98). GPX4 dependency and ferroptosis susceptibility can be acquired by cells in a therapy-resistant cell state (99), as for instance cells undergoing an epithelial-mesenchymal transition (EMT) (100). Hence, targeting leukemic cells via ferroptosis represents a promising new treatment approach, though very few data are available in AML models as yet. Recently, ferumoxytol (Feraheme^©^), an FDA approved intravenous iron nanoparticle for treating iron deficiency in patients with chronic kidney disease, showed anti-leukemic effects *in vitro* and in murine models bearing leukemia cells with low FPN expression (101). Iron salophen complexes, a kind of chemically engineered transition-metal complex, display *in vitro* anti-leukemic properties via ferroptosis or necroptosis (102). Notably, GPX transcription level in AML is associated with a worse prognosis and correlates with the reponse to oxidative stress, inflammation and the immmune response (103). Ferroptosis inducers such as erastin can also enhance the anticancer capacity of conventional chemotherapeutics like cytarabine or doxorubicin (104) in RAS-mutated AML cell lines. Moreover, erastin can boost the activity of more recent compounds such as APR-246, a p53-mutant reactivating compound in cancer (105). Erastin-induced ferroptosis in AML cell lines has been shown to involve high mobility group box 1 (HMGB1) cytosolic translocation, the RAS-JNK/p38 pathway and subsequent TFR1 up-regulation (106). Furthermore, autophagy-mediated ferritin degradation and iron subsequent supply play a key role in ferroptosis, explaining at least in part the anti-tumor activity of dihydroartemisin via the activation of the AMPK-mTOR signaling pathway (92). Recently, iron has shown the capacity to catalyze the oxidative demethylation of protein residues and nucleobases in association as a cofactor of demethylases like PHF8, which is a great interest in AML since demethylating agents have proven efficacy in this context (107). A putative model of the iron metabolism modulation that triggers ferroptosis is provided in Figure 2.

## Conclusion

Recent insights into iron metabolism along with the recent discovery of ferroptosis have opened new avenues in the field of anti-tumor therapies. Metabolism rewiring between glycolysis and mitochondrial oxphos respiration in tumors has proven to be an anchor for new and effective therapeutic combination strategies such as the association of the BCL2-BH3 mimetic and hypomethylating agents, which are able to specifically target the LSC compartment. Additionally, iron metabolism dysregulation toward increased iron demands is a specific feature of tumor cells which has been considered almost exclusively until now as an opportunity to test iron deprivation approaches against the tumor cells, whether than using it as an Achilles heel. Opportunities to tailor new strategies that also utilize ferroptosis will arise in the coming years.

Because non-targeted iron supplementation could increase tumorigenicity and cause deleterious systemic effects, feeding tumor cells with iron or reshaping iron metabolism mechanisms to trigger ferroptosis will be a complex problem to solve. First, because of the heterogeneous sensitivity to iron-catalyzed necrosis among cancers, thorough analyses using *in vitro* and murine AML models are required to assess the feasibility of such strategies. Data are currently lacking in this regard for hematological malignancies and specifically for AML, thus hindering further therapeutic perspectives. Second, in addition to the iron supplementation or iron metabolism actor modulation, combination strategies using ferroptosis inducers are probably desirable to lower the sensitivity threshold of cancer cells to ferroptosis and thus avoid iron-mediated off-target damage. This point is of major interest since great efforts have been made to identify and develop ferroptosis triggers over the past decade. Hence, a widespread introduction of iron-based treatments into the mainstream oncology arsenal in a personalized fashion and with the purpose of inducing iron-catalyzed death is a promising anti-cancer strategy.

## Author Contributions

EG and DB contributed conception and design of the review. RB and NC provided critcial revisions to the content. All authors contributed to the article and approved the submitted version.

## Conflict of Interest

The authors declare that the research was conducted in the absence of any commercial or financial relationships that could be construed as a potential conflict of interest.

## Abbreviations

AA: arachidonic acid
AdA: adrenic Acid
ACSL4: acyl-CoA synthetase long chain family member 4
AML: acute myeloid leukemia
DMT1: divalent Metal (Ion) Transporter 1
FTH: ferritin
FPN: ferroportin
GPX4: gluthatione peroxydase 4
GSH: glutathione
HSC: hematopoietic stem cell
IRE: iron responsive element
IRP: iron regulatory protein
LIP: labile iron pool
LOX: lipoxygenase
LPCAT: lysophosphatidylcholine acyltransferase 1
LSC: leukemic stem cell
NCOA4: nuclear receptor coactivator 4
PE: phosphatidylethanolamine
PL: phospholipids
PUFAs: polyunsaturated fatty acids
ROS: reactive oxygen species
Tf: transferrin
TFR1: transferrin receptor 1.
